# BOSC 2023, the 24th annual Bioinformatics Open Source Conference

**DOI:** 10.12688/f1000research.143015.1

**Published:** 2023-12-07

**Authors:** Nomi L. Harris, Christopher J. Fields, Karsten Hokamp, Jérémy Just, Radhika Khetani, Jessica Maia, Hervé Ménager, Monica C. Munoz-Torres, Deepak Unni, Jason Williams

**Affiliations:** 1 Lawrence Berkeley National Laboratory, Berkeley, California, 94720, USA; 2 Carver Biotechnology Center, University of Illinois Urbana-Champaign, Urbana, Illinois, 61801, USA; 3 Smurfit Institute of Genetics, Trinity College of Dublin, Dublin, D02 PN40, Ireland; 4 Ecole Normale Superieure de Lyon, Lyon, Auvergne-Rhône-Alpes, 69364, France; 5 Bioinformatics Core, Harvard T.H. Chan School of Public Health, Cambridge, Massachusetts, 02115, USA; 6 BD Technologies and Innovation, Research Triangle Park, North Carolina, 27709, USA; 7 Institut Pasteur, Paris, 75015, France; 8 University of Colorado Anschutz Medical Campus, Aurora, Colorado, 80045, USA; 9 Swiss Institute of Bioinformatics, Basel, 4051, Switzerland; 10 Cold Spring Harbor Laboratory, Cold Spring Harbor, New York, 11724, USA

**Keywords:** bioinformatics, open source, open science, open data

## Abstract

The 24th annual Bioinformatics Open Source Conference ( BOSC 2023) was part of the 2023i conference on Intelligent Systems for Molecular Biology and the European Conference on Computational Biology (ISMB/ECCB 2023). Launched in 2000 and held yearly since, BOSC is the premier meeting covering open-source bioinformatics and open science.

Like ISMB 2022, the 2023 meeting was a hybrid conference, with the in-person component hosted in Lyon, France. ISMB/ECCB attracted a near-record number of attendees, with over 2100 in person and about 900 more online. Approximately 200 people participated in BOSC sessions.

In addition to 43 talks and 49 posters, BOSC featured two keynotes: Sara El-Gebali, who spoke about “A New Odyssey: Pioneering the Future of Scientific Progress Through Open Collaboration”, and Joseph Yracheta, who spoke about “The Dissonance between Scientific Altruism & Capitalist Extraction: The Zero Trust and Federated Data Sovereignty Solution.” Once again, a joint session brought together BOSC and the Bio-Ontologies COSI. The conference ended with a panel on Open and Ethical Data Sharing.

As in prior years, BOSC was preceded by a CollaborationFest, a collaborative work event that brought together about 40 participants interested in synergistically combining ideas, shaping project plans, developing software, and more.

## Introduction

The 24th annual Bioinformatics Open Source Conference, BOSC 2023, took place in Lyon, France, as part of this year’s Intelligent Systems for Molecular Biology and European Conference on Computational Biology meetings, known as ISMB/ECCB. Occurring annually since 2000, BOSC has been part of ISMB on all but two occasions. BOSC’s parent organization is the Open Bioinformatics Foundation (OBF), a non-profit, volunteer-run organization that promotes open-source software and Open Science in the biological research community.

**Figure 1.**
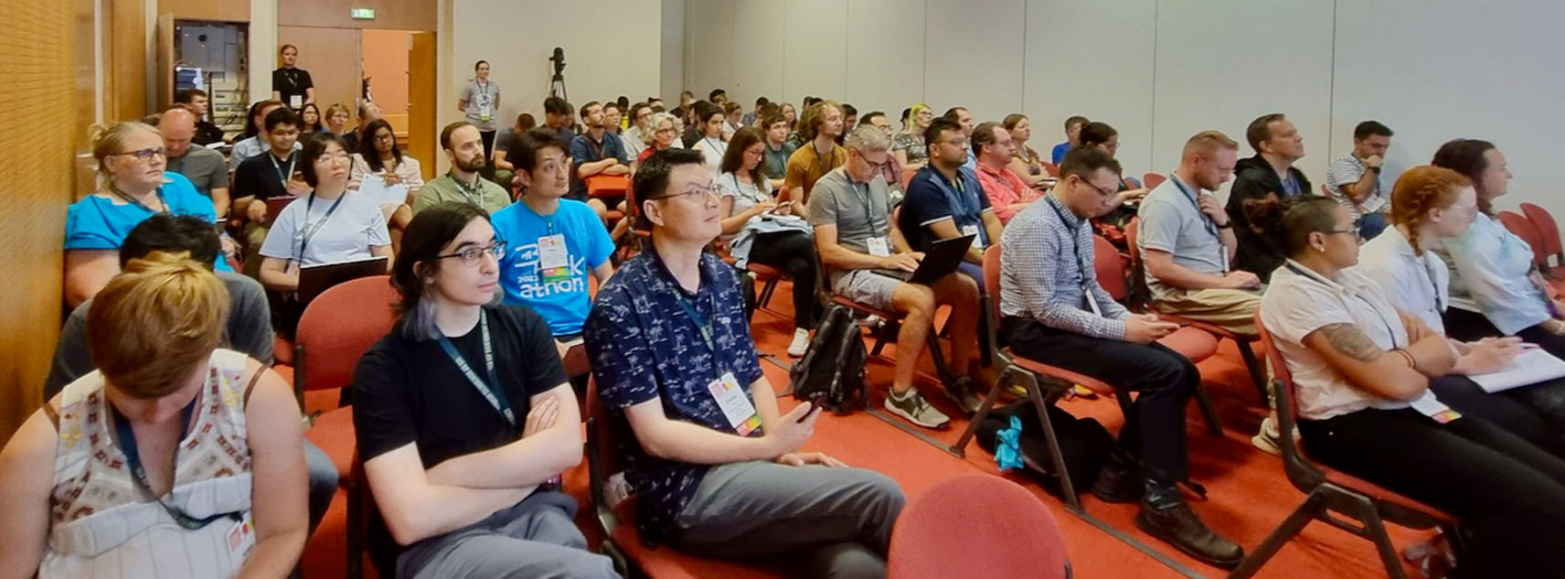
A full room at BOSC 2023 in Lyon, France. BOSC, Bioinformatics Open Source Conference.

Like ISMB 2022, ISMB/ECCB 2023 was a hybrid conference, with the in-person component hosted in Lyon, France. This year’s conference attracted a near-record number of attendees for ISMB, with over 2100 in person and about 900 more online. Approximately 200 people participated in BOSC sessions ( Figure 1).

**Figure 2.**
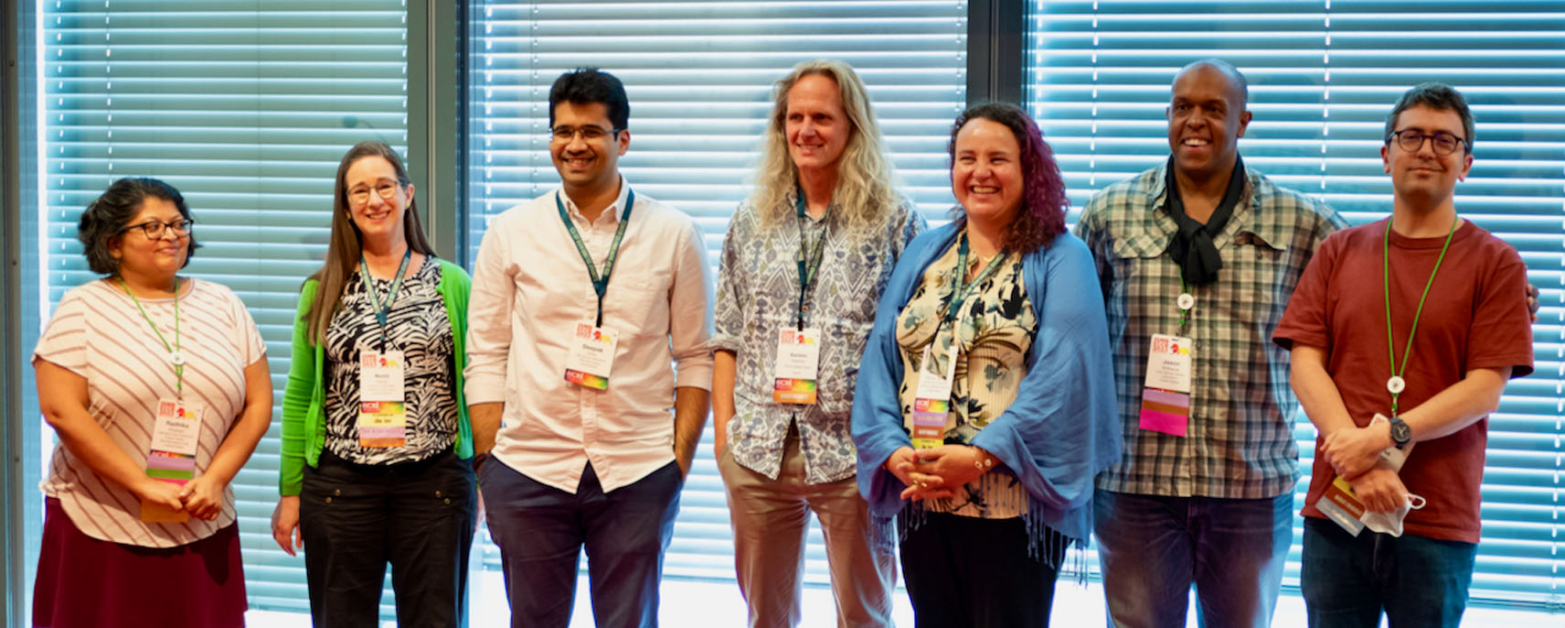
Most of the BOSC organizing committee. From left: Radhika Khetani, chair Nomi Harris, Deepak Unni, Karsten Hokamp, Monica Munoz-Torres, Jason Williams, Hervé Ménager (missing: Jessica Maia and Chris Fields). BOSC, Bioinformatics Open Source Conference.

Unlike many meetings of similar scale, BOSC is entirely volunteer-run. Nomi Harris, a program manager at the Lawrence Berkeley National Laboratory, has chaired BOSC since 2012. She leads an organizing committee that typically includes 7-9 people. This year’s members were Chris Fields, Karsten Hokamp, Radhika Khetani, Jessica Maia, Hervé Ménager, Monica Munoz-Torres, Deepak Unni, and Jason Williams ( Figure 2). In addition to the organizing committee, a review committee of about 30 people thoughtfully reviewed all submitted abstracts, with three reviewers assessing each abstract. Documentation about our review process and rubric has been public since 2020. We strive to provide fair and constructive criticism on all submissions.

## Conference Program

### Keynotes

**Figure 3.**
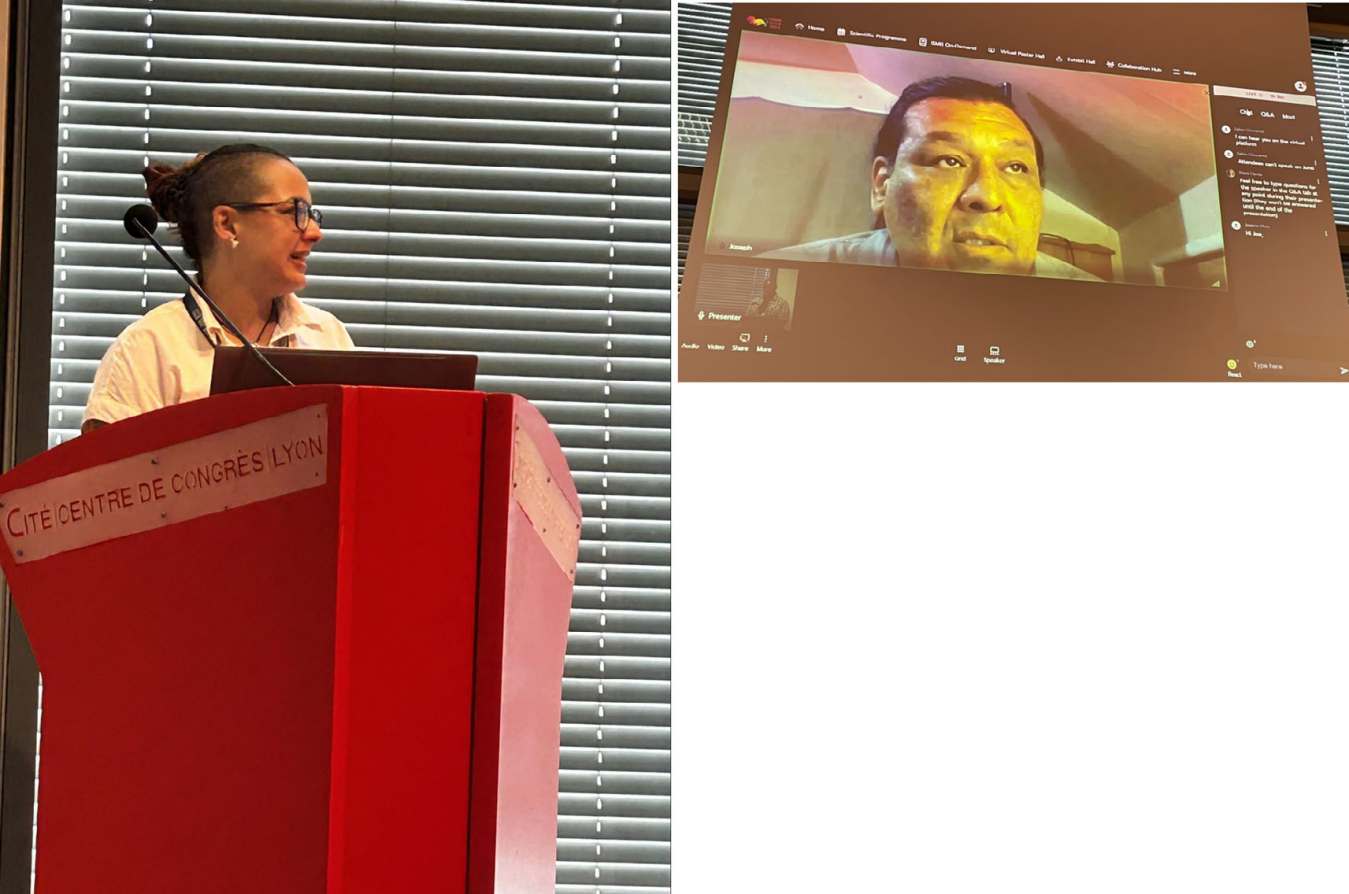
BOSC 2023 keynote speakers Sara El-Gebali (left) and Joseph Yracheta (right). BOSC, Bioinformatics Open Source Conference.

The first BOSC 2023 keynote was delivered by Sara El-Gebali of the SciLifeLab-DataCentre-Sweden, who spoke inspiringly on the topic “A New Odyssey: Pioneering the Future of Scientific Progress Through Open Collaboration,” with case studies showing how open collaboration can strengthen inclusive scientific communities and vice-versa. For example, the fact that 95.5% of genomic research participants in genome-wide association studies (GWAS) are people of European heritage has led to the development of drugs that don’t work for most people in the world. Dr. El-Gebali made many points that resonated with the BOSC audience, such as the need to revamp research reward systems to consider open science practices, not just one-dimensional publication metrics ( Figure 3).

Joseph Yracheta offered a thought-provoking keynote talk entitled “The Dissonance between Scientific Altruism & Capitalist Extraction: The Zero Trust and Federated Data Sovereignty Solution”. Dr. Yracheta is the Executive Director of the Native BioData Consortium, the first 501(c)(3) nonprofit research institute led by Indigenous scientists and tribal members in the United States. He critically examined the relationship between open data and the need for community trust, engagement, and data sovereignty. Naturally, we’re passionate about open science, but Dr. Yracheta’s talk exposed the ways data can be exploited when it’s not connected to collective benefit and informed consent. He shared how Native BioData Consortium is realizing their CARE principles through their work.

### Panel

**Figure 4.**
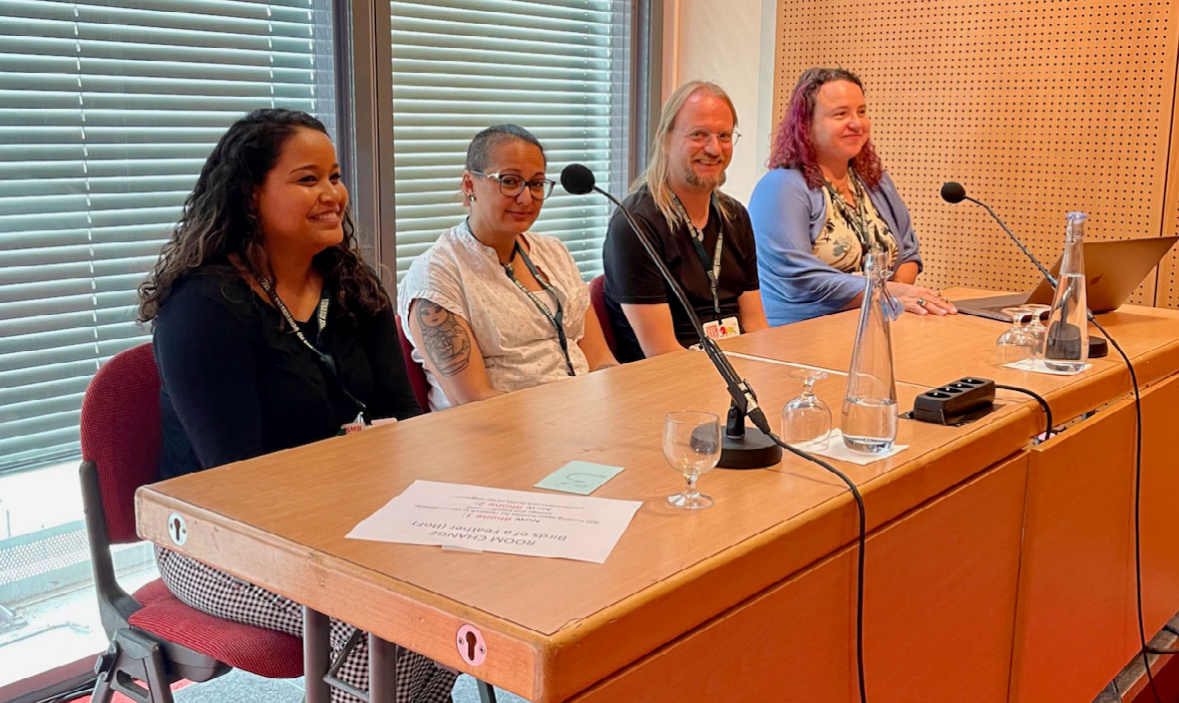
The panel on Open and Ethical Data Sharing featured (from left to right) panelists Verena Ras, Sara El-Gebali, Bastian Greshake Tzovaras, and Joseph Yracheta (not shown in photo), with moderator Monica Munoz-Torres.

BOSC 2023 also featured a panel on Open and Ethical Data Sharing ( Figure 4). Our two keynote speakers (Sara El-Gebali and Joseph Yracheta) plus Verena Ras, who works with international organizations to develop training models that overcome the challenges experienced by developing countries, and Bastian Greshake Tzovaras, a leader in organizing citizen science projects joined moderator Monica Munoz-Torres in the panel. The topics expanded upon some of the points the keynote speakers made, including the observation that there’s no published ethical code for bioinformaticians and the idea that we, individually, and in our scientific societies, must be advocates for better practices in ethical data sharing.

### Talks and posters

**Figure 5.**
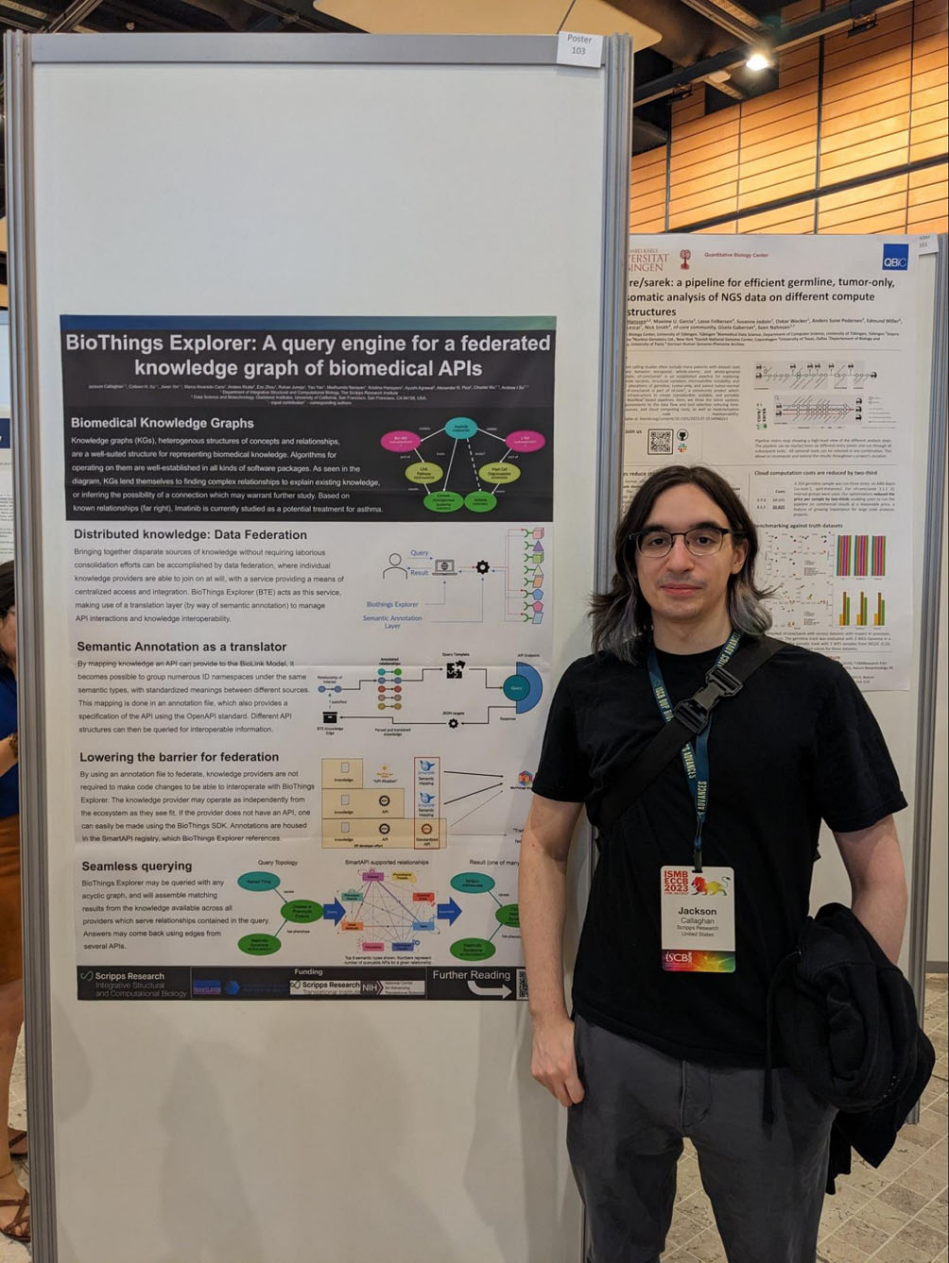
Jackson Callaghan’s poster at BOSC 2023. BOSC, Bioinformatics Open Source Conference.

As usual, BOSC included topical sessions with a total of 53 short and long talks chosen from submitted abstracts. Overall, 49 participants presented their posters in Lyon ( Figure 5) and/or using the online platform. The complete lists of talks and posters can be found at www.open-bio.org/events/bosc-2023/bosc-2023-schedule.

This year’s session topics were:

• Translational bioinformatics
• Workflows
• Data analysis and visualization
• Artificial intelligence (AI) and machine learning (ML)
• Open Science
• Joint session, BOSC + Bio-Ontologies: Standards and frameworks for open science
• FAIR (Findability, Accessibility, Interoperability, and Reuse) and open data

We were pleased to have sufficient submissions this year to put together a translational session covering clinical applications for bioinformatics. Traditionally, BOSC has focused on biological applications, with limited forays into more clinical research; this is at least in part due to the challenges of introducing open approaches into a sphere that is heavily committed to proprietary tools, while still protecting private patient data. Talks in this session included open-source approaches to identifying promising drug targets, preparing medical claims data for research, genetic variant interpretation, and epidemiological surveillance.

For the first time, BOSC included a session on AI/ML. This was a hot topic across many of the tracks at ISMB/ECCB, but most of the approaches covered in other tracks relied on proprietary tools; at BOSC, we shined a spotlight on open approaches.

**Figure 6.**
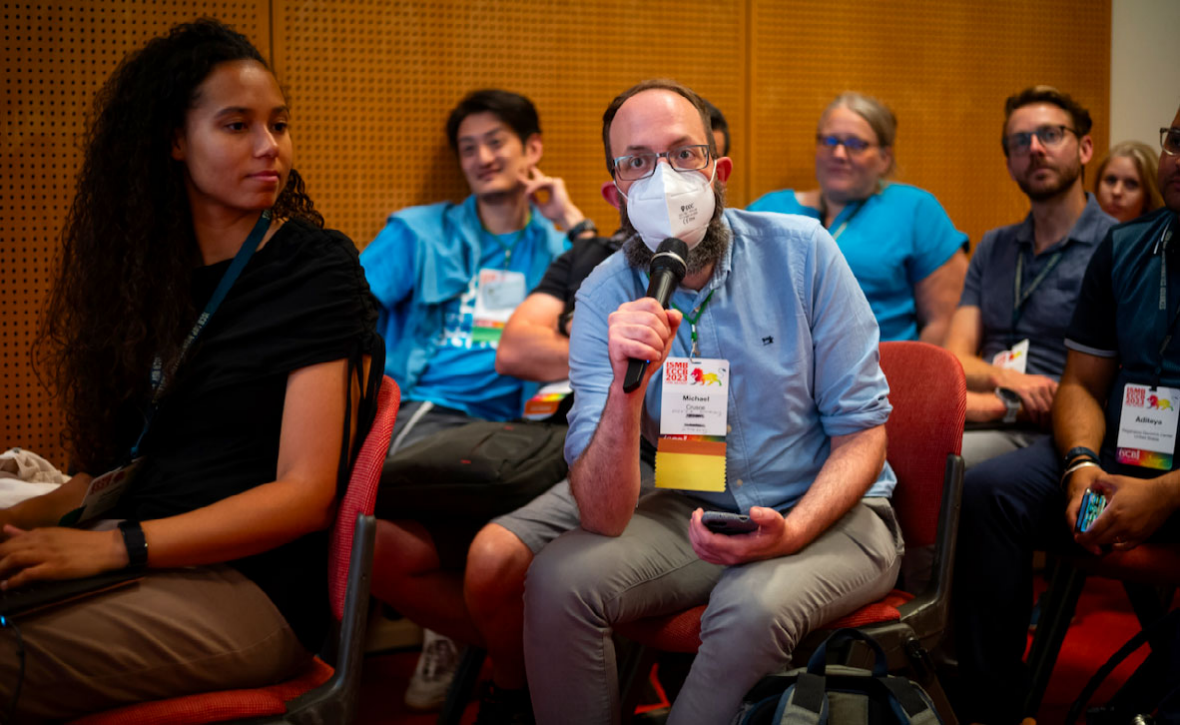
BOSC 2023 participant Michael Crusoe asks a question. BOSC, Bioinformatics Open Source Conference.

The ever-popular workflows session ( Figure 6) covered the whole stack, from infrastructure to FAIR data to UIs. The session on data analysis and visualization has been a mainstay at BOSC since our first meeting; this year’s offerings included updates on established tools such as JBrowse and newer projects such as higlass-python and Multiplayer IGV. The FAIR and Open Data session set the stage for the closing panel. By contrast, a session on Open Science reflected the breadth of BOSC topics, including talks relating to open infrastructures and ecosystems, citizen science, training, outreach, and reproducibility. A well-attended joint session brought together BOSC and the Bio-Ontologies COSI for talks relating to standards (including ontologies) and frameworks for open science.

### CollaborationFest

**Figure 7.**
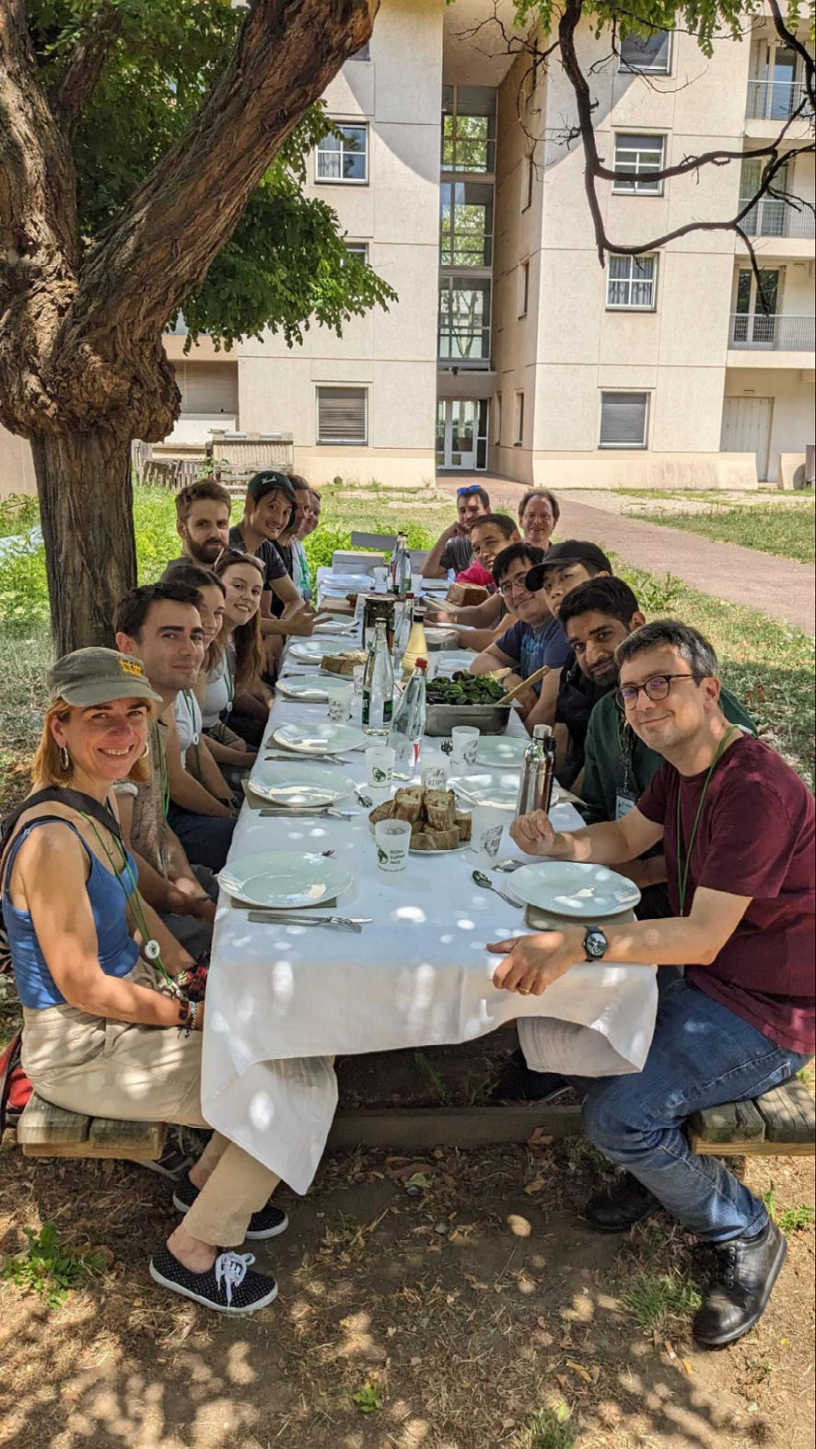
Lunch at CoFest 2023. CoFest (short for CollaborationFest) is a collaborative work event that follows or, in this case, precedes BOSC.

CollaborationFest, CoFest for short, is a collaborative work event that brings together experts in fields as diverse as plant biology and personalized medicine. CoFest has been held immediately before or after BOSC, continuously over the past 13 years. Hervé Ménager and Jérémy Just co-organized CoFest 2023, held the two days before BOSC 2023 in Lyon, France. The event was hosted by the nearby École Normale Supérieure de Lyon. 29 people participated in person ( Figure 7), and others participated online. Thanks to our local hosts, many local and first-time attendees were able to join us. Figuring out how best to integrate remote participants with in-person ones is still challenging.

As in previous years, CoFest participants were involved in a wide variety of projects focused on topics such as the documentation of existing software ( *e.g.*, Biopython tutorial, update of the BLAST Book), the review and discussion of novel technologies ( *e.g.*, Large Language Models), the performance improvement of single-cell RNA-seq data processing (MultiK package), the implementation of new features in existing protein visualization software (iCn3D viewer), and several FAIR-related projects (workflows, development guidelines, provenance, and ontologies). In total, the participants worked on 10 different projects, with tangible accomplishments for several subtasks of these projects. In addition, cross-project discussions facilitated progress on many projects, exchanging perspectives and pointers between participants. Notably, one of the projects that was worked on at CoFest has already led to a peer-reviewed publication: “ Making Biomedical Research Software FAIR.”

### BOSC as a Community of Special Interest (COSI)

Although the BOSC meeting happens only once a year, the conference and its organizers remain actively involved year-round. The ISCB (which organizes ISMB) calls its sub-meetings or tracks “Communities of Special Interest” (COSIs) to capture this ongoing aspect. BOSC is the flagship event organized by the Open Bioinformatics Foundation, a non-profit, volunteer-run organization that promotes open-source software and Open Science in the biological research community. Both OBF and BOSC maintain active social networks year-round, including Slack, Twitter/X (though we are transitioning away from that platform), and Mastodon. The BOSC Slack workspace, which sees heavy use before and during the annual meetings, remains active in between, with members sharing information relevant to open-source bioinformatics and asking and answering each other’s questions.

**Figure 8.**
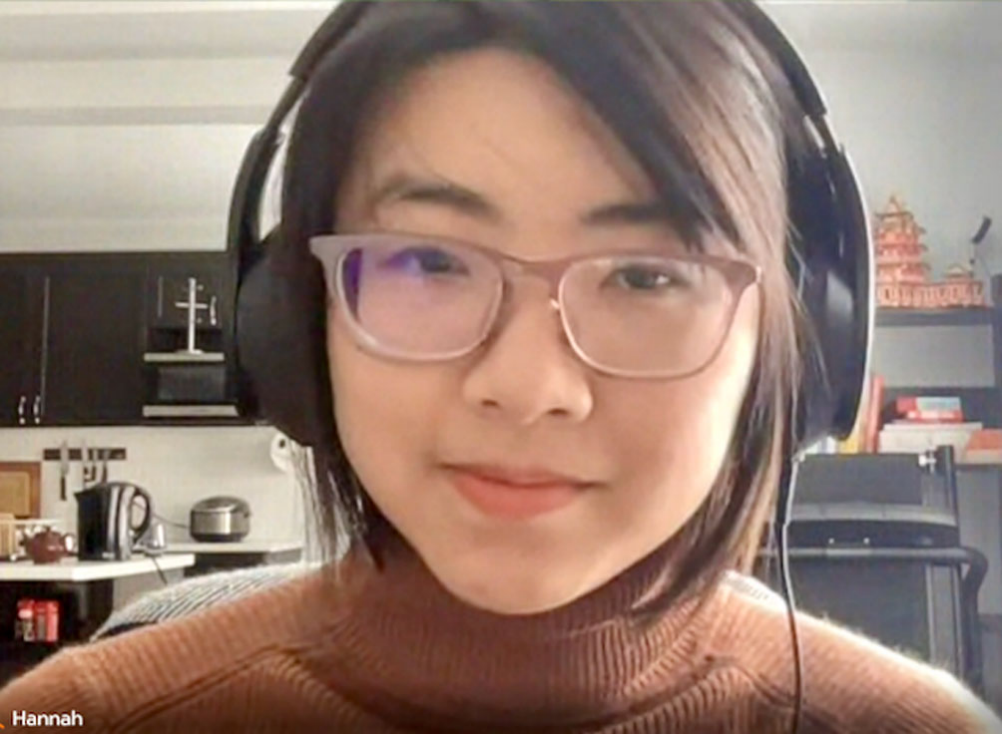
ISCBacademy speaker Hannah Wei, co-founder at the Patient-Led Research Collaborative, during her ISCBacademy talk in February 2023.

BOSC also participates in the ISCB’s free webinar series, called ISCBacademy. In our first ISCBacademy webinar of 2023, Long COVID sufferer and advocate Hannah Wei ( Figure 8) presented Lessons from the Patient-Led Research Collaborative. Our second ISCBacademy of the year, co-sponsored with the Bio-Ontologies COSI, features Sierra Moxon speaking about “LinkML: an open data modeling framework, grounded with ontologies.”

### Diversity, Equity and Inclusion

We put substantial effort and resources into making BOSC more diverse and accessible (see our new DEI page for more info). Three years ago, we added a checkbox to the BOSC abstract submission form to make it easy for authors to request conference registration fee support (these requests are not seen by reviewers). This initiative is funded by sponsorships. Since introducing this option, we’ve offered free ISMB registration to dozens of participants, most of whom are from groups underrepresented at ISMB/BOSC. In 2023, 15 people (13 from underrepresented groups) were granted free registration thanks to a combination of these registration fee waivers and OBF event fellowships.

We have worked to make our keynote speaker selection process inclusive and transparent. In the first phase, we invite as broad a community as possible to nominate potential speakers. Then, we apply our Invited Speaker Rubric to narrow down the list of candidates and solicit community feedback. In particular, we want to know if there are any concerns regarding specific individuals. This process has helped us choose keynote speakers whom we believe meaningfully represent a diversity of backgrounds and ideas. In recognition that not everyone is privileged enough to gift their time, we started offering honoraria to keynote speakers in 2021. As described above, our 2023 keynote speakers discussed topics related to inclusion and equity, as did this year’s panel on Open and Ethical Data Sharing.

**Figure 9.**
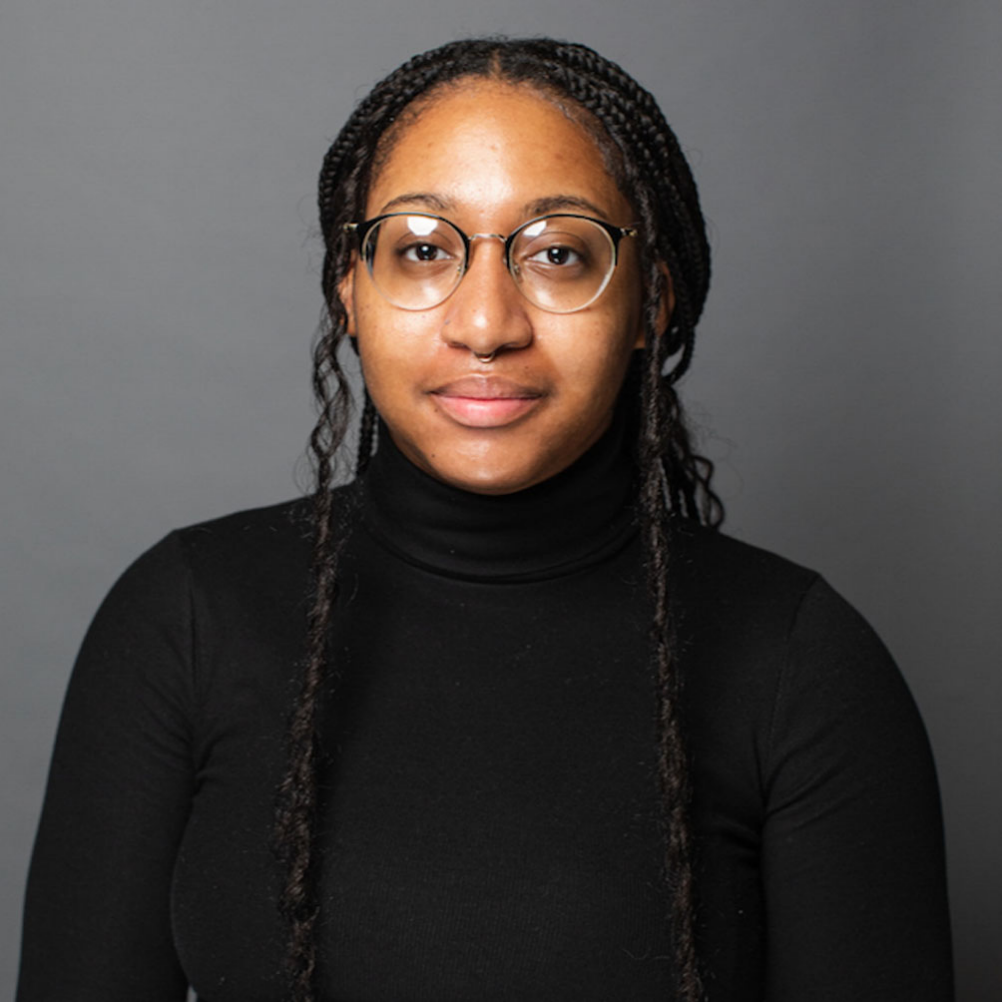
Jenea Adams, panelist in our 2022 “Inclusion & Open Science” panel. Her recent blog post described how BOSC fosters collaboration and inclusivity.

Inclusion offers mutual advantages: when we give a wider range of people a voice, they benefit from being included, and we, in turn, benefit from their contributions. Jenea Adams ( Figure 9), who was a panelist for our 2022 “Inclusion & Open Science” panel, shared her thoughts in a recent blog post, commenting: “BOSC not only showcased the remarkable strides made in computational biology but also emphasized the power of collaboration and inclusivity. Through my participation in the panel on Building and Sustaining Inclusive Open Science Communities, I witnessed the true potential of harnessing diverse perspectives to drive innovation and create a sustainable foundation for open science.”

## Consent

All identifiable subjects in the photos were contacted, and they consented to have their photos used in this report.

## Data Availability

No data are associated with this article.

